# 

**Published:** 1845-09

**Authors:** 


					5 Tol II. ,
SEPTEMBER, 1845.
No. 1.
JO'
or
j' ::
PUBLISHED
Slilsgi
PUBLISHED UNDER THE AUSPICES OF THE
- v ' ? V ? : .,r. . " ? ; < ..."
UARTERLY,*
SocfetB oJ Bcntal Jfcurscons.
EDITO*#:
[ARRIS M.D..D.D.S. a
MAYNARD, M. D., D. D.S.
AMOS WE STCOTT, M. DD. D . S.
ARMSTRONG fc.BEKRY
WOODS, PRINTER.
5 00 per Bnnam, payable in advance.

				

